# Pro-fibrotic compounds induce stellate cell activation, ECM-remodelling and Nrf2 activation in a human 3D-multicellular model of liver fibrosis

**DOI:** 10.1371/journal.pone.0179995

**Published:** 2017-06-30

**Authors:** Vincenzo Prestigiacomo, Anna Weston, Simon Messner, Franziska Lampart, Laura Suter-Dick

**Affiliations:** 1 University of Applied Sciences Northwestern Switzerland, School of Life Sciences, Muttenz, Switzerland; 2 University of Basel, Department of Pharmaceutical Sciences, Basel, Switzerland; 3 InSphero AG, Schlieren, Canton of Zürich, Switzerland; National Institutes of Health, UNITED STATES

## Abstract

**Background & Aims:**

Currently most liver fibrosis research is performed *in vivo*, since suitable alternative *in vitro* systems which are able to recapitulate the cellular events leading to liver fibrosis are lacking. Here we aimed at generating a system containing cells representing the three key players of liver fibrosis (hepatocyte, Kupffer cells and stellate cells) and assess their response to pro-fibrotic compounds such as TGF-β1, methotrexate (MTX) and thioacetamide (TAA).

**Methods:**

Human cell lines representing hepatocytes (HepaRG), Kupffer cell (THP-1 macrophages) and stellate cells (hTERT-HSC) were co-cultured using the InSphero hanging drop technology to generate scaffold-free 3D microtissues, that were treated with pro-fibrotic compounds (TGF-β1, MTX, TAA) for up to 14 days. The response of the microtissues was evaluated by determining the expression of cytokines (TNF-α, TGF-β1 and IL6), the deposition and secretion of ECM proteins and induction of gene expression of fibrosis biomarkers (e.g. αSMA). Induction of Nrf2 and Keap1, as key player of defence mechanism, was also evaluated.

**Results:**

We could demonstrate that the multicellular 3D microtissue cultures could be maintained in a non-activated status, based on the low expression levels of activation markers. Macrophages were activated by stimulation with LPS and hTERT-HSC showed activation by TGF-β1. In addition, MTX and TAA elicited a fibrotic phenotype, as assessed by gene-expression and protein-deposition of ECM proteins such as collagens and fibronectin. An involvement of the antioxidant pathway upon stimulation with pro-fibrotic compounds was also observed.

**Conclusion:**

Here, for the first time, we demonstrate the *in vitro* recapitulation of key molecular and cellular events leading to liver fibrosis: hepatocellular injury, antioxidant defence response, activation of Kupffer cells and activation of HSC leading to deposition of ECM.

## Introduction

Liver fibrosis and cirrhosis are canonical endpoint of many chronic liver diseases, including virus infections (HBV, HCV), non-alcoholic steatohepatitis or damage due to alcohol consumption [1]. In addition, liver fibrosis is also a relevant toxicological outcome and has been identified as an Adverse Outcome Pathway (AOP), a novel tool in human risk assessment designed to provide mechanistic representation of critical toxicological effects [2,3]. Liver fibrosis is characterized by an accumulation of fibrillar extracellular matrix (ECM), leading to liver failure, portal hypertension, and increased risk of cancer. The pathophysiology of fibrosis requires chronic liver damage (including chronic alcohol consumption, chemically-induced hepatocyte damage, and viral infections) and involves the interplay of several hepatic cell types; it requires hepatocyte injury and cell death, activation of Kupffer cells (KC), activation of hepatic stellate cells (HSC), and chronic inflammation [4,5].

Hepatic stellate cells, activated by fibrogenic cytokines (e.g. TGF-β1 and TNF-α), have been identified as the major collagen-producing cells in the injured liver. Stimuli initiating stellate cell activation derive from injured hepatocytes and neighbouring KC. Upon hepatocyte injury, activated KC produce large amounts of reactive oxygen species (ROS) and release cytokines such as TNF-α, TGF-β1, PDGF and IL1β, leading ultimately to stellate cell activation and increased deposition of fibrillar components of the ECM [4–6]. Activated stellate cells, in turn, produce more TGF-β1 and potentiate and perpetuate their activation in an autocrine loop [7].

It is well documented that liver diseases including hepatitis, fibrosis, cirrhosis, and hepatocellular carcinoma induce antioxidant stress response [8]. Oxidative stress also contributes to the release of pro-fibrogenic growth factors, cytokines and prostaglandins that may lead to liver fibrosis and/or cirrhosis [8]. Nrf2 (NF-E2-related factor-2) is an essential transcription factor that regulates an array of detoxifying and antioxidant defence and is finely regulated also by its interaction with Keap1 [9]. Yang et al. showed up-regulation of Keap1 and Nrf2 mRNA and protein in liver tissues of CCl4-induced fibrosis of rat compared with tissues of wild type animals [10].

Emerging anti-fibrotic therapies aim at inhibiting the accumulation of fibrogenic cells and/or preventing the deposition of ECM proteins [4]. The advances in the research of anti-fibrotic therapies are however hampered by the lack of appropriate *in vitro* systems for the study of liver fibrosis. Until now, the majority of the investigations on liver fibrosis are still performed in rodents that underwent chemically-induced fibrosis [11]. These animal models have the advantages of providing the physiological relevance, but with the strong disadvantages of being time consuming, expensive as well as non-human. The efficient development of anti-fibrotic drugs will therefore strongly depend on the availability of a suitable *in vitro* system that more faithfully replicates the pro-fibrogenic microenvironment of human liver [5].

Biological relevant models to study liver fibrosis require functional hepatocytes, as well as KC and HSC in a quiescent (non-activated) status and in close spatial interaction. Three-dimensional (3D) cell culture systems appear to outperform conventional cell cultures with regards to their metabolic activity and responses to toxicants [12,13]. Several methods have been published for the generation of scaffold-free liver MT; however, these systems are generally based on primary cells and often underrepresent non-parenchymal cells [14,15]. Also, most *in vitro* liver models are of limited use due to short longevity in culture, inadequacy of cell composition and/or high handling complexity [13,14,16–18]. Recently, work on a fibrotic 3D-model based on hepatocytes and HSC has been published, but this system lacks macrophages as a key component in the chain of events leading to fibrosis [18]. A suitable *in vitro* model for the study of liver fibrosis should mimic processes that involve the relevant cell types (hepatocytes, KC and HSC) leading to the development of the fibrotic phenotype. Such a system would be a major asset for the understanding of the pathophysiology of fibrosis, as well as for the experimental evaluation of substances with regards to the pro-fibrotic and anti-fibrotic potential.

Here, we aimed at investigating liver fibrosis mechanisms in a novel human hepatic microtissue (MT) model that responds to pro-fibrotic stimuli in a similar way to what is known to occur in the human liver. To this end, we utilized human relevant cell lines representing the hepatocyte, the KC and the HSC. With this system, we could not only recapitulate HSC- and KC activation and other key molecular events implicated in fibrosis, but also the deposition of ECM *in vitro*. Our results show that methotrexate (MTX) and thioacetamide (TAA), two well characterized compounds that cause hepatic fibrosis in animals and man [19, 20] elicited the fibrotic phenotype *in vitro*. This system can thus be used for the investigation of cellular and molecular events involved in the development of fibrosis as well as an *in vitro* test system for the evaluation of anti-fibrotic therapies.

## Material and methods

### Reagents and chemicals

Cell culture media for HepaRG cells were purchased from Biopredic. DMEM High Glucose (Cat. 41965) and Fetal Bovine Serum (FBS) (Cat. 10270) were purchased from Invitrogen. Penicillin-Streptomycin (Cat. A8943) used for cell culture was purchased by AppliChem). LPS (Cat. L3129), TNF-α (Cat. SRP3177), TGF-β1 (Cat. T5050), Thioacetamide (TAA) (Cat. 163678) and Methotrexate (MTX) (Cat. M8407) were purchased from Sigma.

### Human cell lines

HepaRG cells were obtained from Biopredic International (Rennes, France). The cells were seeded at 1 x 10^5^ undifferentiated cells/cm^2^ in ADD710 Growth Medium Supplement (Biopredic). The cells were cultured at 37°C under 5% CO_2_ for 14 days before differentiation. After 14 days of culture, cell differentiation was induced with ADD720 Differentiation Medium Supplement (Biopredic) for 14 days. Then the cells were maintained for up to 4 weeks.

hTERT-HSC were kindly provided by Dr. Bernd Schnabl (UC San Diego, USA) [21] and were cultured in DMEM High Glucose supplemented with 10% FBS and 1% Penicillin-Streptomycin. The cells were kept in the humidified incubator at 37°C with 5% CO_2_.

THP-1 monocytic cells (Cell Line Service) were cultured at 2–10 x 10^5^ cells/mL in RPMI 1640 containing 10% FBS, 1% Penicillin/Streptomycin and maintained at 37°C under 5% CO_2_. THP-1 cells were differentiated into macrophages over 48 hours in RPMI 1640 medium containing 5–25 ng/mL PMA, according to a previously published method [22]. The supernatants were then removed and the wells were washed with fresh medium. Successful differentiation was assessed by the ability of the cells to secrete cytokines upon exposure to 1 μg/mL LPS for 48h.

### Immunocytochemistry analysis

Monolayer cultures of hTERT-HSC were fixed in 4% paraformaldehyde (PFA) for 15 minutes, followed by permeabilization with 0.1% Triton-X-100 for 20 minutes. Blocking was performed with 1% BSA in PBS for 60 minutes and washing with PBS; all steps were performed at RT. Primary antibody against αSMA (Sigma, A5228, dilution 1:200) and secondary antibody Alexa Fluor^®^ 488 F(ab')2 Fragment of Goat Anti-Mouse IgG (H+L) (Invitrogen, A11017, dilution 1:400) were used for the staining.

### Generation of microtissues

All microtissues (MTs) were generated using the GravityPLUS^™^ Hanging Drop System from InSphero (Cat. ISP-06-001). Briefly, 40 μL cell suspension/well were pipetted in a 96-well-format GravityPLUS^™^ plate that has been designed specifically to generate reproducible hanging drops. In this system, cells are allowed to assemble themselves in a scaffold-free manner forming spherical MTs. After 2–3 days incubation, MTs are transferred to 96-well GravityTRAP^™^ plates, were they can be maintained in culture over several weeks. Human liver MTs were generated using 2'000 cells per MT, with either HepaRG-cells (hepatocyte monoculture), or HepaRG in combination with THP-1 and hTERT-HSC. Cell-ratios were empirically chosen to give the best resemblance of the native cellular distribution in liver, approximately 80% hepatocytes and 20% NPCs (including inflammatory cells and stellate cells), as assessed by immunohistochemistry.

### Pharmacological stimulation of microtissues

For functional characterization, the generated MT were exposed to LPS (2 μg/mL), TGF-β1 (1 ng/mL), or TNF-α (50 ng/mL). Further, MTs were exposed to the pro-fibrotic compounds MTX (30–250 μM) and TAA (10–80 mM). The supernatants were collected for protein analysis and the MTs were used for viability assay (MTT and ATP), mRNA extraction or immunohistological analysis.

### Immunohistochemistry

MTs were fixed with 4% PFA 1h in PBS containing calcium and magnesium. Fixed microtissues were embedded in 2% agarose in PBS. The samples were subjected to paraffin embedding, cut and analysed with standard procedures. Microtissues were stained with standard hematoxylin & eosin (H&E) and for immunohistochemistry with the following antibodies: albumin (BETHYL Laboratories INC, Cat. A80-229A), Ki67 (Novocastra Laboratories, Cat. NCL-L-Ki67-MM1), vimentin (Epitomics, Cat. 2707–1), αSMA (Sigma, Cat. F3777), Collagen I (Abcam, Cat. ab88147) and CD68 (Novacastra Laboratories, Cat. NCL-L-CD68).

### Quantification of immunohistochemistry staining

Five random areas from each immunohistochemistry (IHC) specimen were selected. Staining intensity on each image file was assessed by using the IHC Toolbox on the image analysing software NIH ImageJ (version 2.0.0-rc-56/1.51h). Two different colour segmentations were used: one recognized brown-positive cells and the other blue-positive area (nuclei). Integrated optical density was obtained as total number of brown pixels multiplied by the brown intensity of those pixels, and quantitative IHC staining value (QISV) was calculated as integrated optical density divided by total area occupied by the brown (positive cells to the staining) and blue cells (total cell number) [23].

### Cell viability assay

The viability of the microtissues was assessed by using the Cell-Titer Glo^®^ Luminescent Cell Viability Assay 2.0 (Promega), following standard laboratory procedures.

For some MTs the viability was also determined by 3-(4,5-dimethylthiazol-2-yl)-2,5-diphenyltetrazolium bromide (MTT) assay. Briefly, 0.5 mg/mL MTT solution in medium was added to each well and incubated at 37°C for 4 hours. The medium was then replaced by 89 μL DMSO, incubated on a shaker for a few minutes; 11 μL Sörensen buffer was added to each well and the absorbance was measured at 550 nm.

### Gene expression analysis

mRNA was isolated following TRIzol extraction procedure. RNA was reverse transcribed using a reverse transcriptase (Promega) and oligo dT (Qiagen) and real time PCR was performed using FastStart TaqMan Mix (Roche) and TaqMan probes from Invitrogen. Real time, TaqMan PCR was performed on selected genes (Col1α1, Col4α1, fibronectin1, CD44, IL6, TGF-β1, αSMA, Nrf2 and Keap1) (see Table 1). The following qRT-PCR Program was used: 10 minutes denaturation at 95°C, followed by 40 cycles of 15 seconds at 95°C and 1 minute at 60°C. The Ct values were assessed using the Corbett Rotorgene Analysis Software 6000 and actin was used as an internal standard for the normalization of the fold changes of each gene of interest (GOI).

**Table 1:** TaqMan probes used for the research.

Gene of interest	Abbreviation	Invitrogen Ref.nr.
Actin Beta (Housekeeping gene)	ACTB	Hs99999903_m1
Collagen 1 alpha 1	COL1α1	Hs00164004_m1
Collagen 4 alpha 1	COL4α1	Hs00266237_m1
Fibronectin 1	FN1	Hs00415006_m1
CD44 (hyaluronic acid receptor)	CD44	Hs01075861_m1
Interleukin 6	IL6	Hs00985639_m1
Transcription growth factor Beta 1	TGF-β1	Hs00998133_m1
Actin, alpha 2, smooth muscle	ACTA2 (αSMA)	Hs00909449_m1
Nuclear factor (erythroid-derived 2)-like 2	NFE2L2 (Nrf2)	Hs00975961_g1
Kelch-like ECH-associated protein	Keap1	Hs00202227_m1

### ELISA

The presence of cytokines (TNF-α) and extracellular matrix components (COL1α2 and MMP2) in the supernatants was determined using commercial ELISA kits: human TNFα (Thermo Fisher, KHC3014), COL1α2 and MMP2 (Cloud Clone Corp., SEA215Hu and SEA100Hu).

### Statistical analysis

Data were analysed using GraphPad Prism 7 (GraphPad Software, inc.) and expressed as mean values ± SD or mean values ± S.E.M. The Student *t* test was used for comparison between two groups. Data from three or more groups were analysed by one-way analysis of variance with Tukey's multiple comparisons test. IHC analysis was performed in 5 independent areas/staining, significance was calculated using Student *t* test. *P* < 0.05 was considered to be significant.

## Results

### Characterization of hTERT-HSC

**Fig 1:**
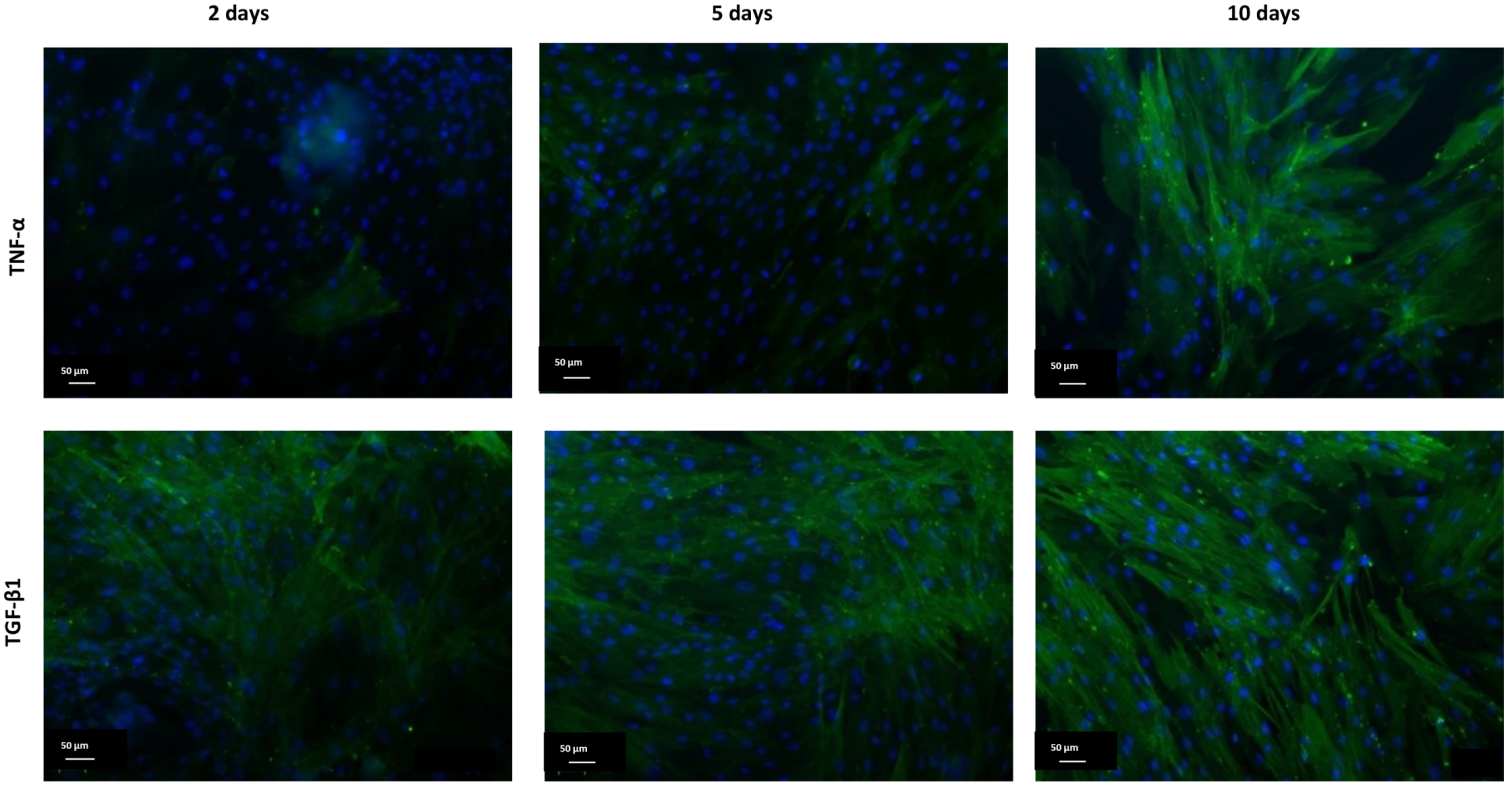
TNF-α and TGF-β1 promote αSMA production in monolayer culture of hTERT-HSC. hTERT-HSC cells were treated for 2, 5 or 10 days with TNF-α (50ng/mL) or TGF-β1 (1ng/mL). After treatment, the cells were fixed and stained against αSMA (green) and nuclei (DAPI, blue). Pictures taken using fluorescence microscopy. Scale bar: 50μm.

The hTERT-HSC cell lines showed typical stellate cell morphology with dendritic structure. The cells were highly proliferative and showed low expression of αSMA, a marker for activated stellate cells. Upon treatment with TGF-β1 or TNF-α, activation of hTERT-HSC cells was observed, indicated by enhanced staining for αSMA (Fig 1). TGF-β1 elicited stellate cell activation after 2 days (with an increase of αSMA-positive cells from 10% to 90%), whereas 10-day stimulation was required with TNF-α to elicit a similar phenotype (Fig 1).

### Characterization of macrophages

**Fig 2:**
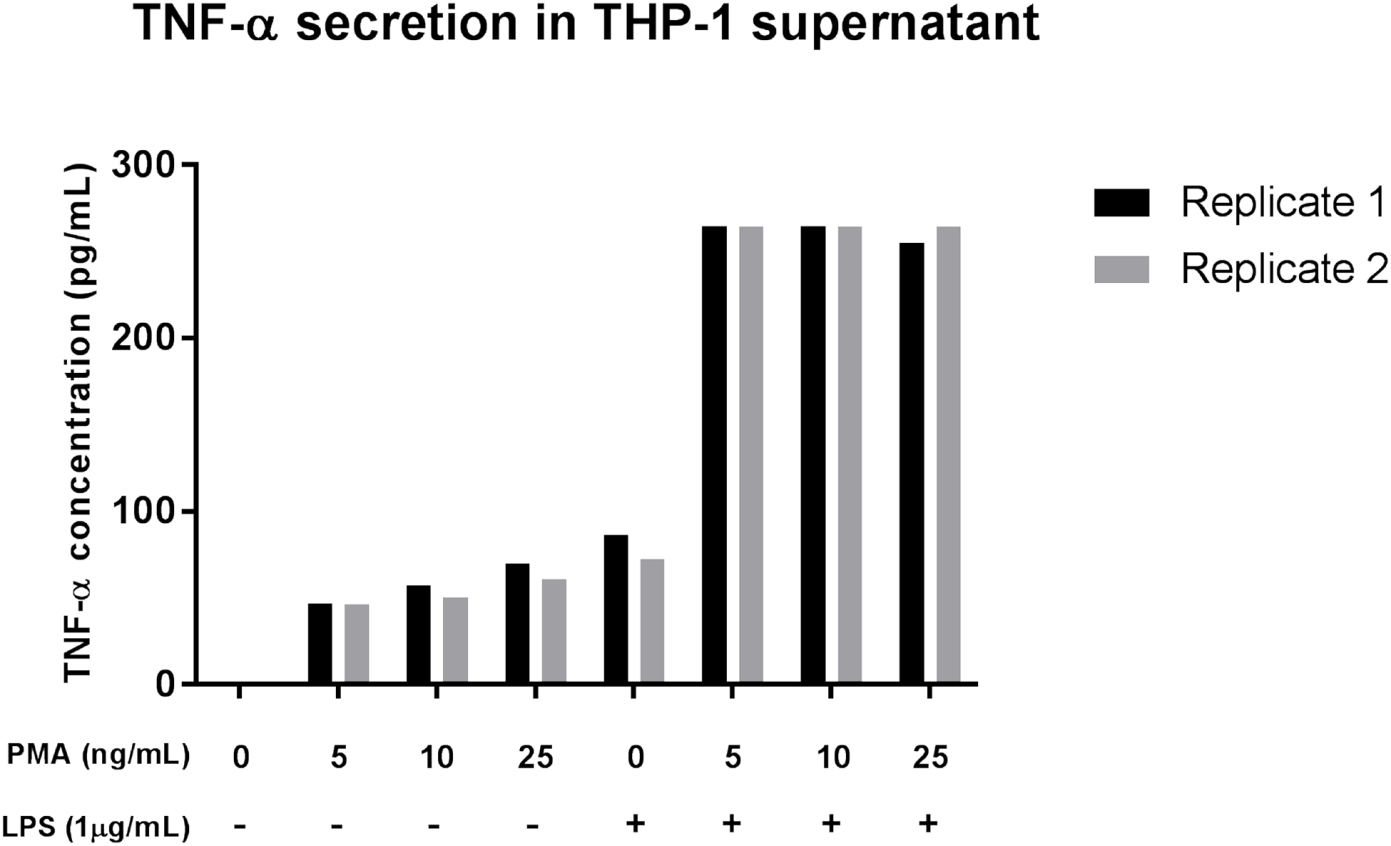
LPS induces TNF-α production in PMA-treated THP-1 cells. THP-1 were treated with 0, 5, 10 and 25 ng/mL PMA for 48h prior exposure to 1 μg/mL LPS. The concentration of TNF-α was measured from the 48h culture supernatants by ELISA. These findings indicate that PMA differentiated THP-1 cells are well differentiated and yet respond adequately to a subsequent low-concentration of LPS. Values are presented as duplicates.

After differentiation of THP-1 monocytes to macrophages with PMA for 48 h about 90% of THP-1 cells attached and spread showing the expected phenotype. Differentiated macrophages exposed to LPS (1 μg/mL) for 48 h showed the expected increase of TNF-α secretion. The level of secreted TNF-α was increased 5-fold by LPS in all the tested samples (Fig 2). 10ng/mL PMA was chosen as minimal concentration for a stable differentiation, since the cells easily detached after PBS washing in the 5 ng/mL PMA samples.

### Morphological characterization of microtissues

**Fig 3:**
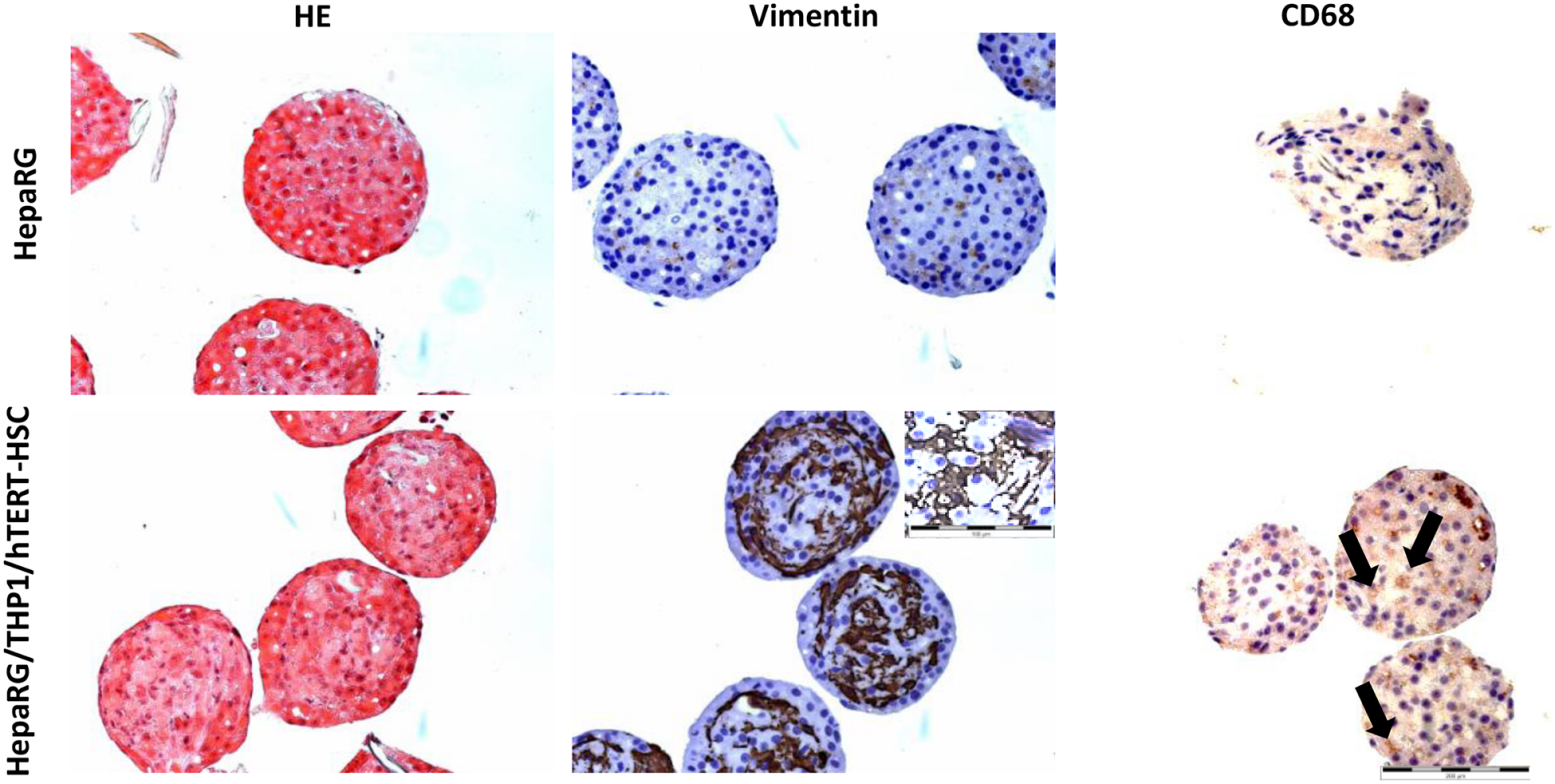
Staining of formalin fixed paraffin embedded human microtissues generated with HepaRG-cells or HepaRGs/THP-1/hTERT-HSC. Microtissues were stained with Hematoxylin & Eosin (H&E), mesenchymal NPC-marker vimentin and macrophages marker CD68. Microtissues were kept in culture during 14 days before performing histological staining. Co-culture systems showed positive staining for vimentin and CD68 indicating the presence of the three different cell types. Arrows indicate CD68-positive cells. Dendritic stellate cells are shown in the zoom of vimentin staining picture. Scale bar: 200μm (20X magnification) and 100μm (40X magnification).

Microtissues (MTs) showed a regular spherical shape and a diameter of approximately 200–350 μm. The presence of the three cell types in the co-cultures was demonstrated by the expression of vimentin, marker of mesenchymal non-parenchymal cells, and CD68, marker for macrophages (Fig 3). In addition, we observed dendritic-shaped cells expressing vimentin, likely to be quiescent hTERT-HSC. The vimentin-positive cells are always located in the inner part of the microtissues, indicating a preferential spatial organization of the three cell types, with hTERT-HSC and macrophages located in the centre and surrounded by the hepatocytes.

### Responses to LPS, TNF-α and TGF-β1

**Fig 4:**
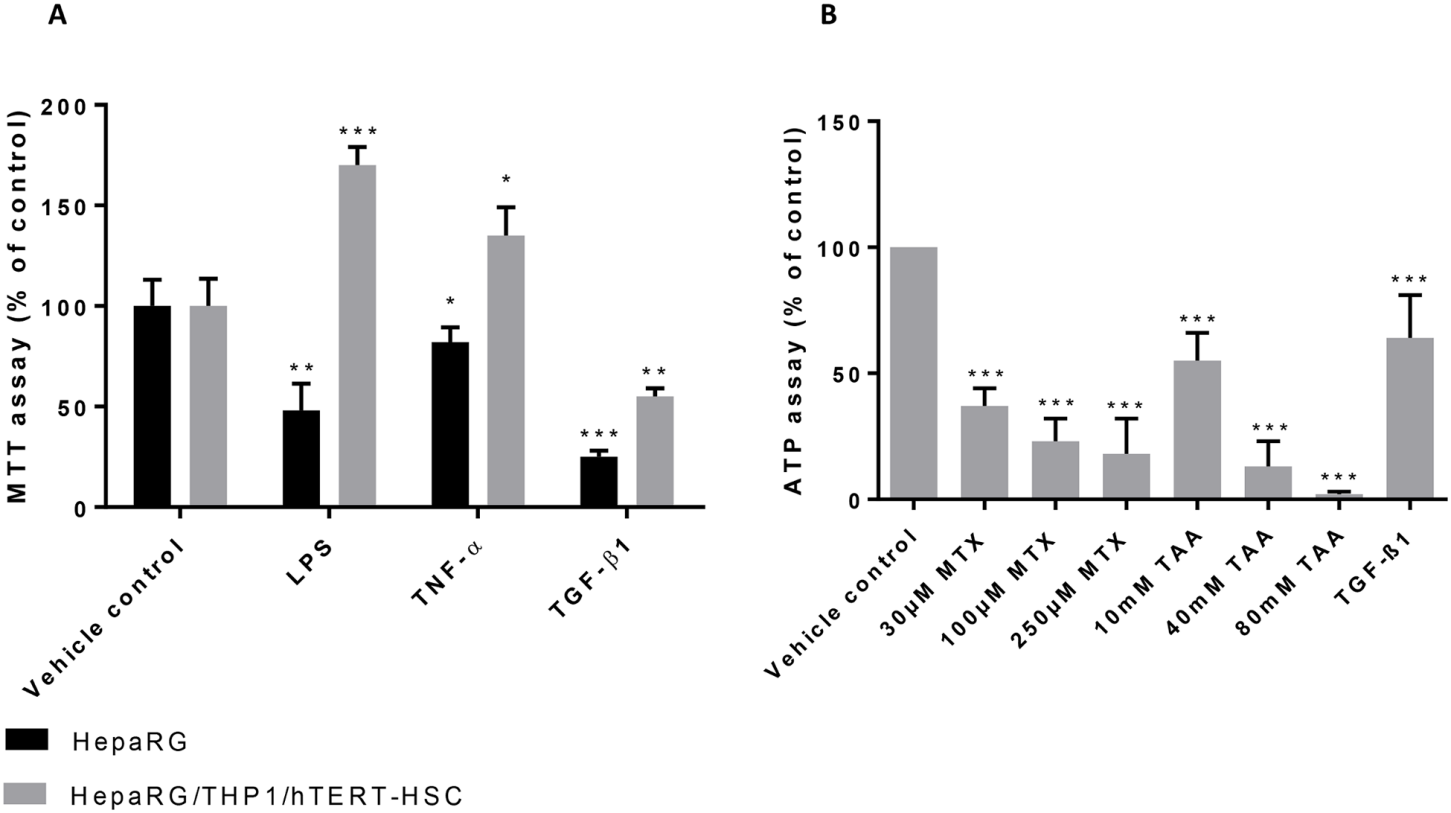
Viability response of human liver microtissues treated with pro-fibrotic compounds. (A) Effect of LPS, TNF-α and TGF-β1 on viability of human liver microtissues was assessed by MTT assay. Microtissues were incubated with 0.5mg/mL MTT solution for 4h after 14 days of exposure to the tested compounds. DMSO and Sörensen buffer were then added into the wells and absorbance was read at 550nm using FlexStation 3. Values are expressed as percentage of negative control. *; P ≤ 0.05, **; P ≤ 0.01, ***; P ≤ 0.001 vs vehicle control. (n = 6, mean ± SD). (B) Effect of MTX, TAA and TGF-β1 on the ATP production. ATP content was measured using CellTiter-Glo^®^ Luminescent Cell Viability Assay 2.0 after 14 days of exposure to MTX, TAA and TGF-β1. Values are expressed as percentage of negative control. ***; P ≤ 0.001 vs vehicle control (n = 6, mean ± SD).

The treatment with LPS and TNF-α resulted in increased cellular viability of co-cultured microtissues, whereas TGF-β1 decreased their viability after 14 days of treatment (Fig 4A). Significant decrease in cell viability was also showed after treatment with LPS and TNF-α in HepaRG-only microtissues, in contrast with the cell proliferation showed in the co-culture model with the same compounds.

**Fig 5:**
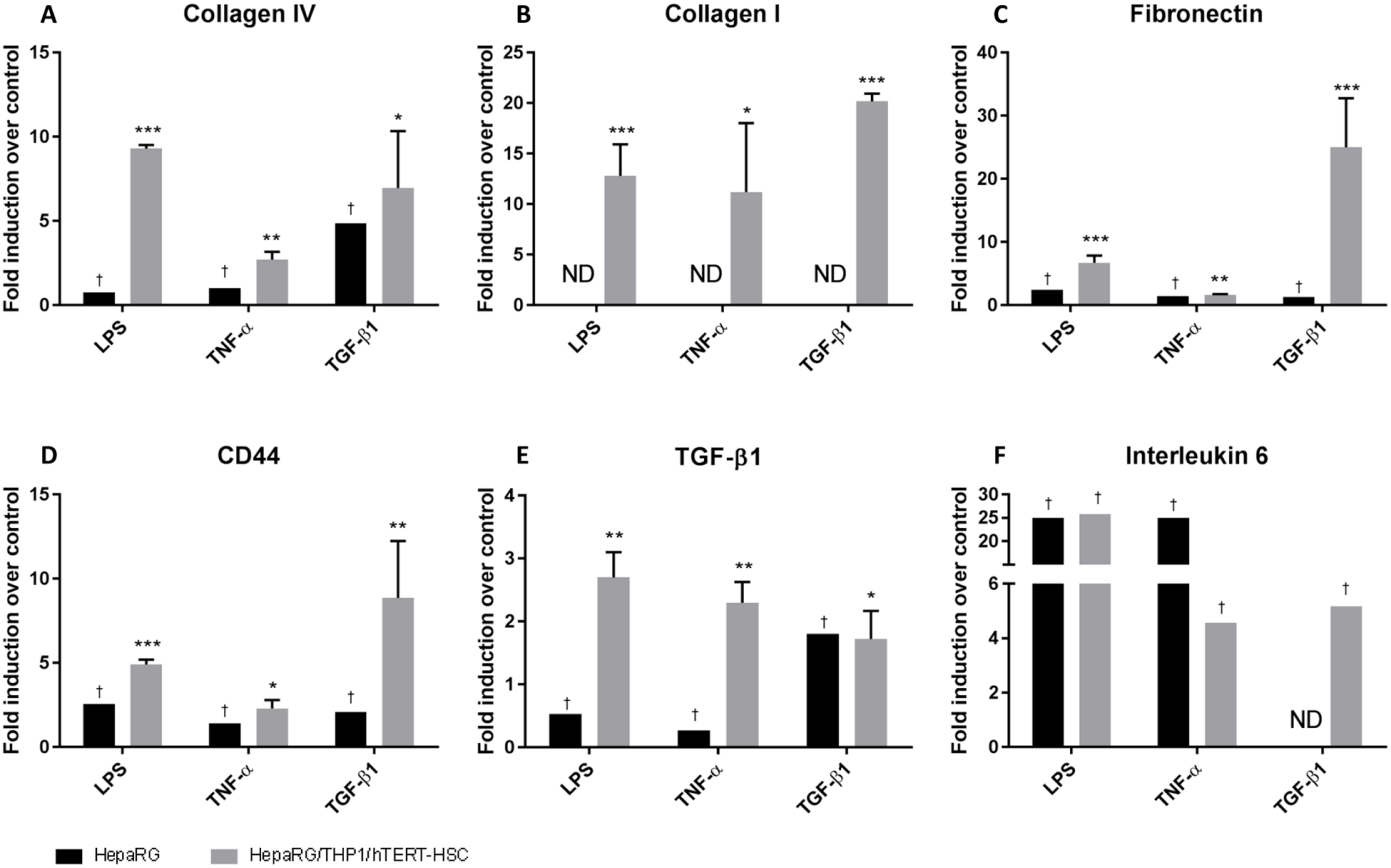
Gene expression of fibrotic markers and cytokines in human liver microtissues exposed to LPS, TNF-α and TGF-β1. mRNA was extracted using TRIzol conventional procedure and fold induction were calculated as 2^(-ΔΔCT) for each sample and vehicle control and expressed as mean fold induction ± S.E.M of three replicates with six MTs each. Actin was used as reference gene for each sample. † pool of 16 microtissues analysed as duplicate; no statistical analysis were performed on these samples. ND: no-detected values. *; P ≤ 0.05, **; P ≤ 0.01, ***; P ≤ 0.001 vs vehicle control.

HepaRG showed a strong induction of IL-6 expression after stimulation with LPS and TNF-α (Fig 5F). On the other hand and as expected, HepaRG monocultures, exhibited only moderate or no increase in expression of extracellular matrix proteins after stimulation with TGF-β1 (Fig 5).

In the co-cultures containing the three cell types, analysis of gene expression changes after treatment with LPS, TNF-α, and TGF-β1 showed a strong transcriptional induction of the extracellular matrix proteins collagen I and IV, fibronectin, CD44, as well as the cytokines IL-6 and TGF-β1 (Fig 5).

### Responses of multicellular MTs to the pro-fibrotic substances MTX and TAA

**Fig 6:**
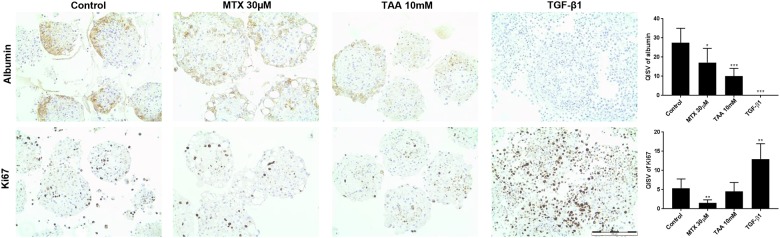
Albumin production and cell proliferation in liver microtissues. Formalin fixed paraffin embedded slides of HepaRG/THP-1 macrophages/hTERT-HSC microtissues were stained with Albumin and Ki67 antibodies after 14 days of treatment with MTX, TAA and TGF-β1. Microtissues were fixed in 4% PFA and embedded in 2% agarose prior paraffinization. Microtissues showed decrease in albumin production after MTX, TAA and especially TGF-β1 exposure. Ki67 shows strong induction of cell proliferation in the microtissues after TGF-β1 exposure. Scale bar: 200μm. Graphics show Quantitative IHC Staining Value (QISV) as mean ± S.D. (N = 5). *; P ≤ 0.05, **; P ≤ 0.01, ***; P ≤ 0.001 vs vehicle control.

**Fig 7:**
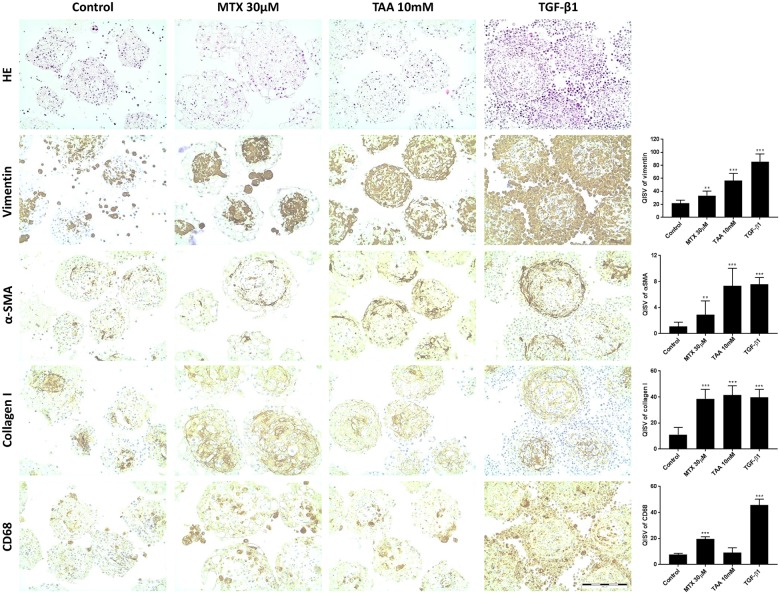
Immunostaining of formalin fixed paraffin embedded human microtissues after exposure to MTX, TAA and TGF-β1. Formalin fixed paraffin embedded slides of HepaRG/THP-1 macrophages/hTERT-HSC microtissues were stained with Hematoxylin & Eosin (H&E), vimentin, α-smooth muscle actin (αSMA), collagen I and CD68 after 14 days of treatment with MTX, TAA and TGF-β1. Microtissues were fixed in 4% PFA and embedded in 2% agarose prior paraffinization. Microtissues showed increase in the vimentin, αSMA, collagen I and CD68 positive cells after MTX, TAA and TGF-β1 exposure. Vimentin and CD68 stainings show proliferation of stellate cells and THP-1 macrophages in the microtissues, suggesting the onset of inflammation process, while αSMA and collagen I indicate activation of stellate cells and deposition of collagen. Scale bar: 200μm. Graphics show Quantitative IHC Staining Value (QISV) as mean ± S.D. (N = 5). *; P ≤ 0.05, **; P ≤ 0.01, ***; P ≤ 0.001 vs vehicle control.

**Fig 8:**
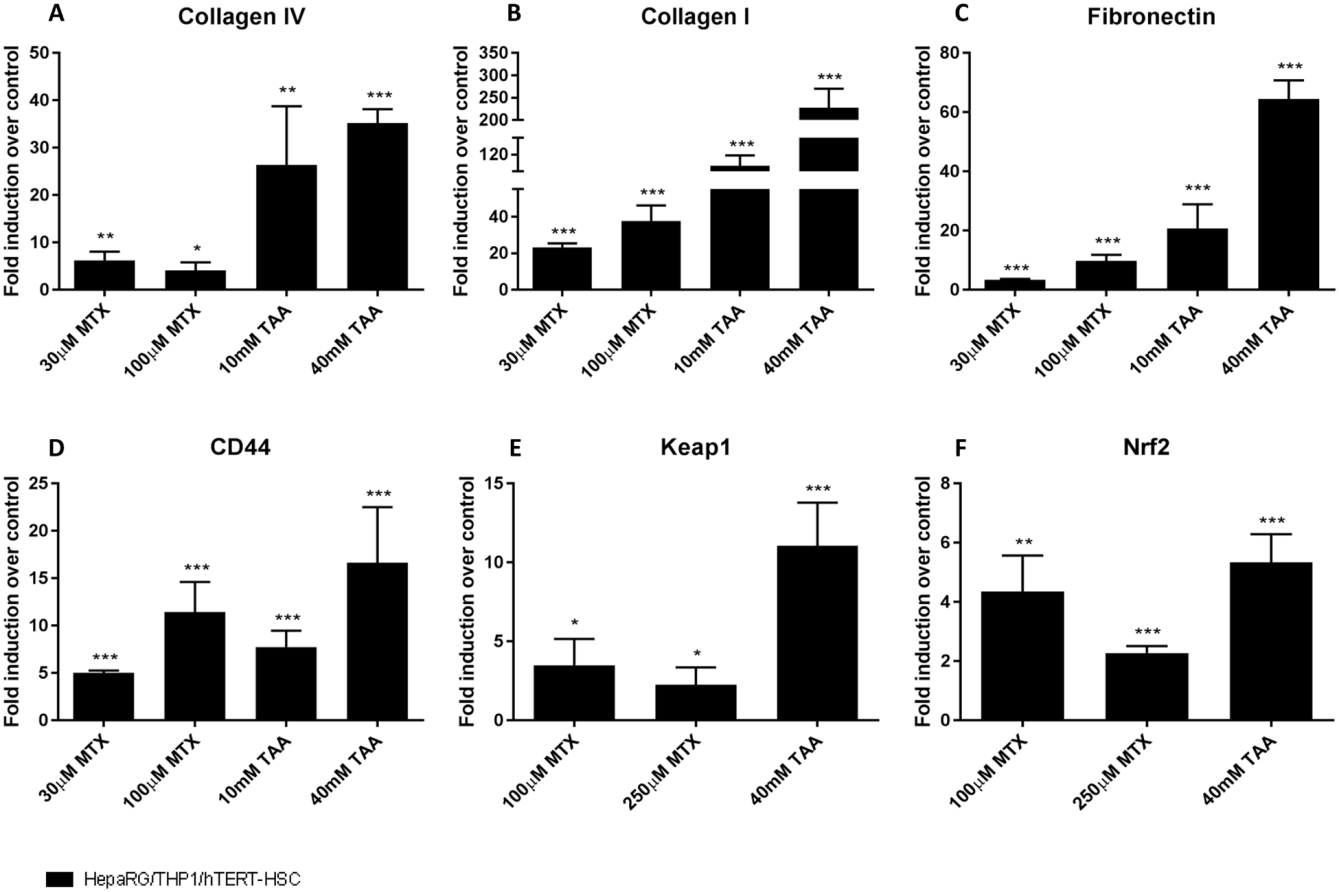
Effect of MTX and TAA on gene expression of fibrotic markers in human liver microtissues. mRNA was extracted using TRIzol conventional procedure and fold induction were calculated as 2^(-ΔΔCT) for each sample and negative control and expressed as mean fold induction ± S.E.M. of three replicates with six MTs each. Actin was used as reference gene for each sample. *; P ≤ 0.05, **; P ≤ 0.01, ***; P ≤ 0.001 vs vehicle control.

In order to test whether the newly established *in vitro* model is capable of recapitulating induction of liver fibrosis, we tested the pro-fibrotic chemicals methotrexate (MTX) and thioacetamide (TAA). Non-treated MTs were rather quiescent and maintained a relatively constant cell composition with minimal proliferation over at least 2 weeks (Fig 6). Exposure of co-culture MTs to MTX and TAA over 14 days showed a dose-dependent decrease in viability (Fig 4B), mainly due to an effect on HepaRG cells. This is consistent with the decrease in albumin staining detected by IHC (Fig 6). After TGF-β1 stimulation, NPCs proliferated, as indicated by an increase of Ki67 and vimentin positive cells; the number of vimentin positive cells showed a 4-fold increase as assessed by QISV (Fig 7). The increase in the CD68-, αSMA- and vimentin-positive cells, clearly show that a change in the cellular composition of the microtissues is occurring after treatment with TGF-β1, MTX and TAA; in particular hTERT-HSC and THP-1 macrophages are proliferating at the expense of the HepaRG, which are decreasing. In addition, the increase in αSMA-positive cells demonstrated the activation of HSC. Thus, the MTs show a strong change in the cellular composition after stimulation with TGF-β1 and, to a lesser extent, with the profibrotic compunds MTX and TAA (Fig 7). The cellular defence mechanism was also activated upon treatment of the MT with TAA and MTX, as the mRNA levels of both Nrf2 and Keap1 were increased (Fig 8).

**Fig 9:**
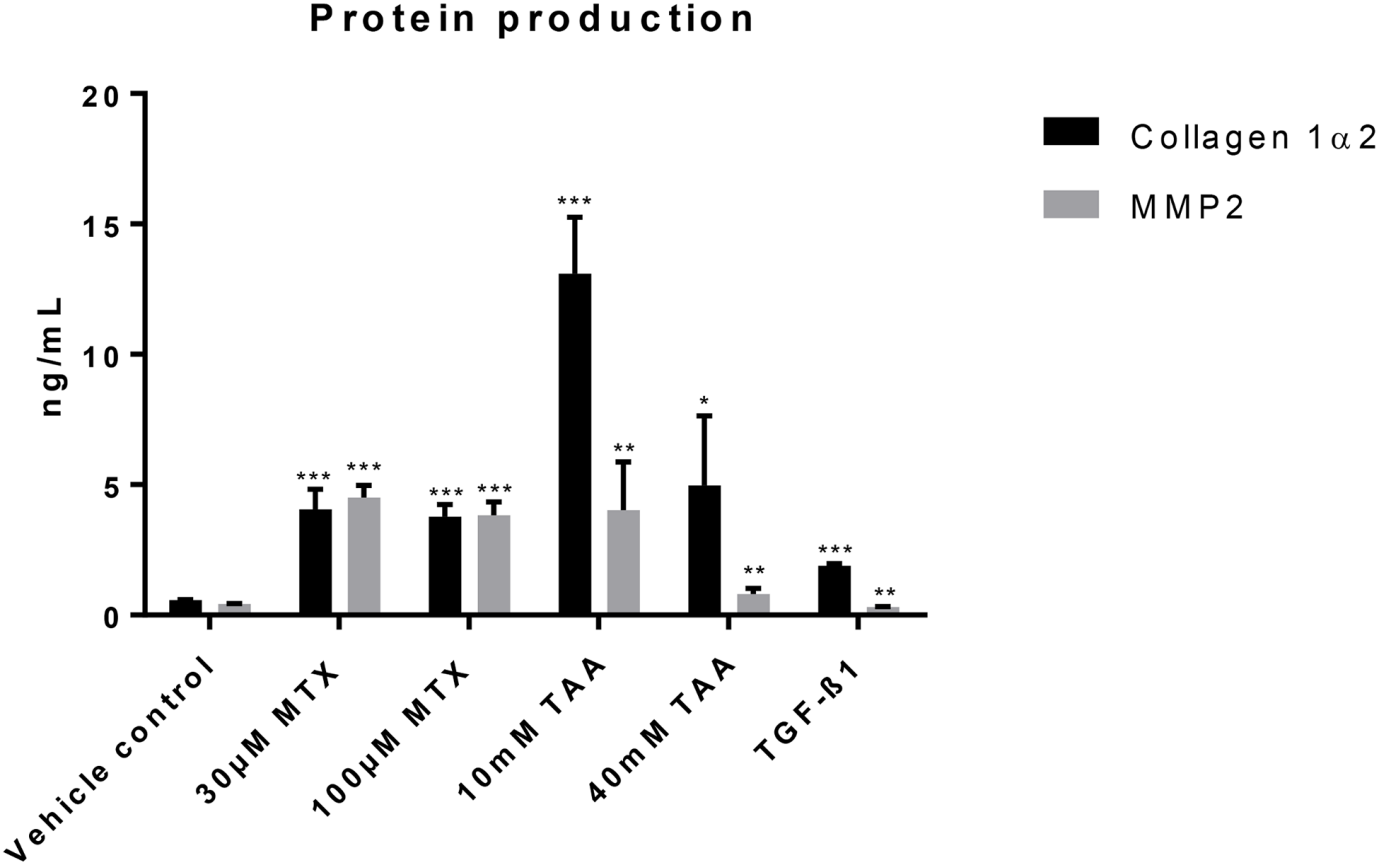
Secretion of Collagen I and MMP2 in supernatant medium after exposure of microtissues to pro-fibrotic compounds. Protein amount was assessed by ELISA in supernatant medium after 14 days of exposure to MTX, TAA and TGF-β1. *; P ≤ 0.05, **; P ≤ 0.01, ***; P ≤ 0.001 vs vehicle control (n = 4, mean ± SD).

Extracellular matrix remodelling expected during the progression of fibrosis was observed in the MTs treated with the model compounds. Gene expression of MTs treated with MTX and TAA showed significant, dose-dependent transcriptional induction of collagen I, collagen IV, fibronectin I and CD44 (Fig 8). The observed increase in gene expression resulted in increased secretion of collagen 1 and MMP2 in the supernatants at 14 days (Fig 9). These findings were further confirmed by the increased protein deposition of collagen I and αSMA observed by histological examination (Fig 7).

## Discussion

In this work, we provide experimental evidence demonstrating that multicellular MTs generated with well characterized human cell lines can recapitulate the key cellular and molecular events leading to hepatic fibrosis. We reproducibly generated human liver MT using HepaRG, hTERT-HSC and THP-1, three human cell lines representing hepatocytes, macrophages and stellate cells. The use of stellate cells in culture has been attempted previously but often not considered a good option for the study of fibrosis *in vitro* due to the fact that stellate cells often undergo spontaneous activation when grown on plastic dishes [24]. This is mainly due to the physical properties of cell culture dishes, which have a tissue tension greater than that of the fibrotic/cirrhotic liver (20 KPa) and much larger than that of normal liver tissue (5 KPa) [5]. hTERT-HSC have been reported to revert to a more quiescent status when cultured on extracellular matrix components [25]. In our hands, however, hTERT-HSC showed low levels of expression of the stellate cell activation marker αSMA before induction with TGF-β1. In both 2D culture and in the scaffold-free 3D MT, hTERT-HSC responded to pro-fibrotic stimuli such as LPS, TNF-α, TGF-β1 and pro-fibrotic compounds (MTX and TAA) by attaining an activated status. In the scaffold-free 3D liver MTs that we generated, the hTERT-HSC are kept in a more physiological environment, surrounded by other relevant cell types (HepaRG and THP1) as shown by IHC staining for αSMA and vimentin. In addition, the co-culture of HSC with hepatocytes and Kupffer cells is a model that incorporates all three cell types involved in the AOP leading to fibrosis, and is therefore very well suited to recapitulate cell-cell interactions leading to fibrosis *in vitro*. The results we obtained after stimulation with TGF-β1 clearly demonstrate that the hTERT-HSC cell line is able to respond to well established pro-fibrotic stimuli by increasing the expression of the key factors: the activation marker αSMA and ECM proteins (collagen I and IV, fibronectin, and CD44). Similarly, in our hands differentiated THP-1 macrophages served as an excellent surrogate for KC, as they were able to produce cytokines (in particular TNF-α) upon stimulation with LPS in both 2D and 3D cultures. In our system, we used HepaRG cells as equivalent of hepatocytes. These cells are known to display many characteristics of human hepatocytes [26] including retention of metabolic activity [27]. Similarly to reported data with primary murine hepatocytes [28], HepaRG MTs responded to LPS and TNF-α by increasing transcription of IL6, showing for the first time that also HepaRG cells are able to produce IL6 following injuring stimuli. HepaRG were also able to produce albumin in the 3D culture, indicating a functional phenotype up to three weeks in culture. However, albumin was significantly decreased after treatments with MTX and TGF-β1 suggesting hepatocytes damage and onset of fibrosis in the microtissues. The higher biological relevance of this model system allows studying responses in an integrated biological system with intricate crosstalks between the main contributing cell types to fibrosis. For example, biological response to LPS stimulation is only possible in presence of inflammation responsive cells (THP-1) and resulted in high activation of HSCs. This would not be possible in conventional monocultures. IHC staining for vimentin (as marker for NPCs) shows a physiological liver cell ratio of Hep/NPCs with approximately 80% hepatocytes, 20% NPCs including inflammatory cells and stellate cells prior treatments. Thus, we were able to generate a complex cellular model system with cell types and ratios which mimic *in vivo* liver organization. Moreover, with such model systems, it is for the first time possible to study in detail the contribution of the different cell types on induction and progression of fibrosis in an *in vitro* model system.

### Human liver MT recapitulate the sequence of events leading to fibrosis

**Fig 10:**
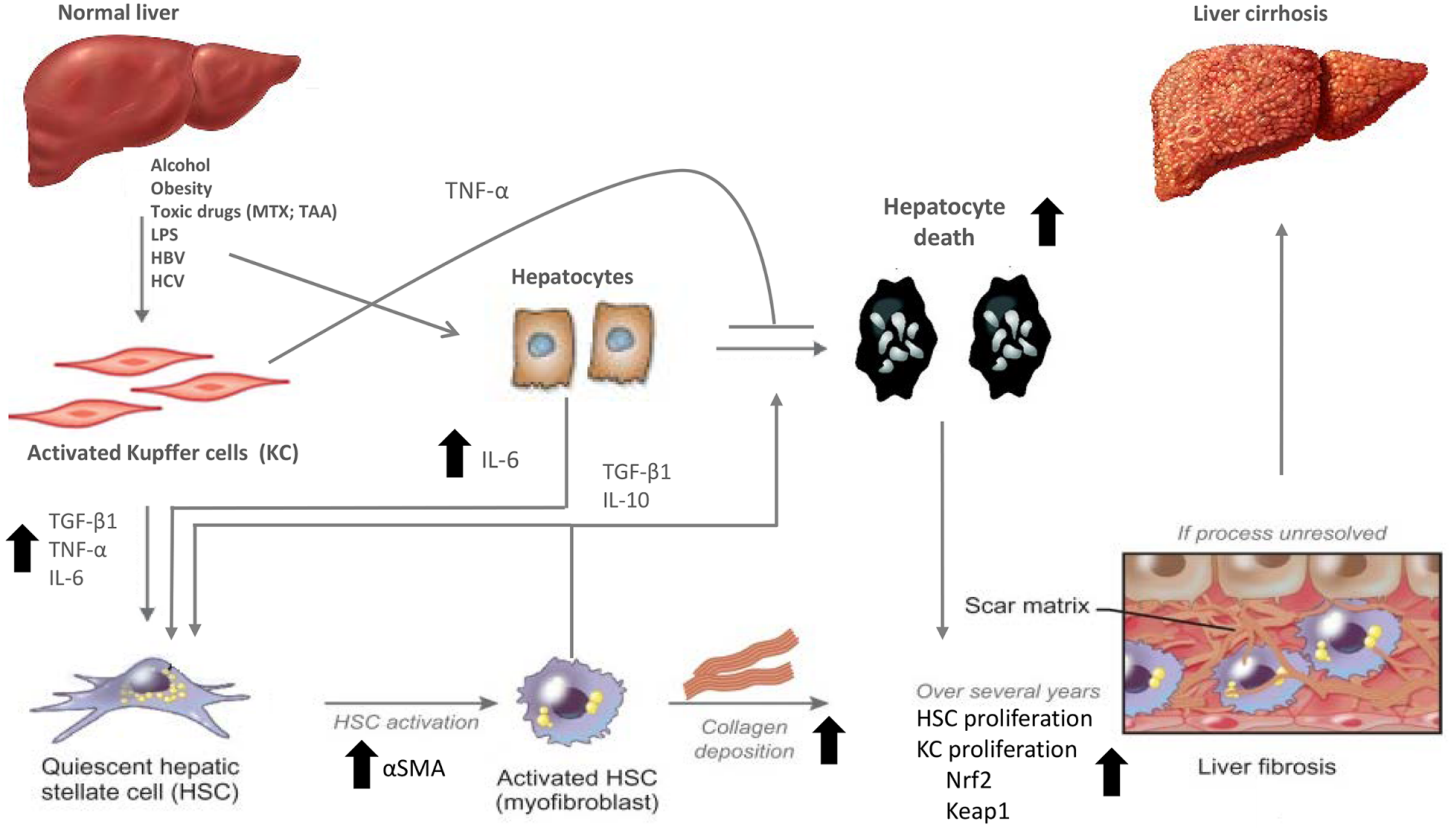
Molecular signalling between hepatic stellate cells, Kupffer cells and hepatocytes after liver injuries. The picture shows the potential complex interactions between matrix-producing hepatic stellate cells and liver-resident macrophages and hepatocytes after liver injuries such as pro-fibrotic compounds (MTX and TAA). Some of these interactions have been previously published, some are supported by our data and some are still speculative. In our liver microtissues THP1 macrophages (as surrogate of Kupffer cells) produced large amount of TNF-α, IL6 and TGF-β1 detected by increase in gene expression. HepaRG (hepatocyte-like cells) also produced cytokines (IL-6) after specific stimulation such as LPS and TNF-α and showed hepatocyte damage after exposure to TGF-β1 as demonstrated by decreased albumin staining. The cytokines were able to enhance stellate cell activation (increase in αSMA production) and collagen deposition. Furthermore increase in expression of oxidative related genes, such as Nrf2 and Keap1, was measured at high concentrations of MTX and TAA suggesting the involvement of this cellular defence. Arrows indicate measured parameters.

The recently published sequence of key events leading to liver fibrosis involve hepatocellular injury/death, KC-activation and macrophage recruitment, TGF-β1 expression and release of cytokines (TNF-α and IL6), HSC activation, and collagen accumulation [29]. In Fig 10, we depicted our results in the context of the published AOP and of literature on known pathways related to liver fibrosis. Our results strongly indicate that the events leading to fibrosis in vivo can be reproduced by the herein described liver MT model system.

First: Hepatocellular injury/cell death was detected after exposure of MTs to TGF-β1, MTX and TAA by cell viability assays where the treatments caused a dose-dependent decrease in viability. Hepatocyte damage was also highlighted by albumin staining with a complete loss of albumin after exposure to TGF-β1. With LPS, TNF-α, TGF-β1 we could demonstrate that the major target of the cytotoxicity in the co-culture system were the HepaRG cells. MTs consisting of HepaRG alone showed a decrease in cell viability, while in co-culture systems there seemed to be an increase in the number of living cells. This suggests compensation due to the proliferation of hTERT-HSC and/or THP-1 macrophages. Immunostaining results (Ki67 and vimentin), confirm the proliferation of vimentin-positive non-parenchymal cells and macrophages (expressing CD68) after TGF-β1 stimulation. HepaRG injury could also be confirmed by upregulation of mRNA level of IL6 after exposure to LPS and TNF-α, suggesting the onset of an inflammatory response.

Second: Hepatic KC and macrophages are usually responsive to the TLR4-ligand LPS by increased cytokine release [30]. After exposure of liver microtissues to LPS, we detected an increase of expression of TNF-α, IL6 and TGF-β1, indicating the onset of an inflammatory response. Moreover, treatment of microtissues with TNF-α, also led to increased expression and secretion of TGF-β1, produced either by the THP-1 cells or by the hTERT-HSC in agreement with a described autocrine loop [31].

Third: The last cellular events in the cascade leading to liver fibrosis involve activation of stellate cells and ECM-remodelling [29]. This was also observed in our system as exposure of MTs to TGF-β1 led to an increase in the number of αSMA-positive cells and the induction of ECM components (collagen I, IV, fibronectin, CD44, and MMP2). CD44 (hyaluronic acid receptor) is usually upregulated in stellate cells of injured liver and it is also correlated to their activated and migratory phenotype [32]. Also MMP2 upregulation in the supernatant confirms the remodelling of the ECM, as previously published [33]. Thus, the co-cultured MTs were able to recapitulate the described cellular key events that lead to liver fibrosis.

### Response of liver microtissues to pharmacologically induced fibrosis

Similar to the results obtained with the positive control TGF-β1, both MTX and TAA induced strong expression of ECM-components and a rearrangement of the cellular composition of the MTs: increased numbers of vimentin-positive NPC cells appeared surrounded by big and round apoptotic cells that could be the injured HepaRG, as also supported by the decrease in albumin. These NPCs contributed to changes in the ECM, demonstrated by the increased secretion and deposition of collagen I, the strong transcriptional induction of hyaluronic acid receptor (CD44) and fibronectin, as well as a 10-fold increase in secretion of MMP2 into the medium. Taken together, the results suggest an increase in the propensity of the stellate cells to become invasive and highly proliferative and demonstrate the remodelling of the ECM upon challenge of liver MT with fibrotic compounds. All these effects were more pronounced in TAA-treated samples than in MTX-treated samples.

In addition to the effects on viability and ECM, the involvement of oxidative stress and the activation of the Nrf2 pathway in liver fibrosis have previously been reported [10]. The induction of Nrf2 and Keap-1 after exposure to MTX and TAA clearly indicates that this cellular defence mechanism is active in our culture system. In the early stages of fibrosis, ROS produced directly by injured hepatocytes and/or recruited neutrophils could directly induce activation of Kupffer cells and macrophages [34]. The Nrf2 and Keap-1 gene upregulation we measured in our system could be related to similar protective mechanisms, but due to the multicellular nature of the co-cultured MT, we cannot pinpoint if this pathway has been activated in the HepaRG cells or in the THP-1 or hTERT-HSC.

## Conclusion

Summarizing, in this study, we have demonstrated that a 3D-liver MT co-culture containing HepaRG, THP-1 and hTERT-HSC is able to recapitulate the known cellular events leading to the fibrotic phenotype [29].

Treatment with pro-fibrotic substances demonstrates that this system reproduces the key cellular and molecular events including hepatocellular injury, activation of the cellular defence pathway, macrophage activation, stellate cell activation and deposition of ECM (Fig 10). Pharmacological interventions (pro-fibrotic compounds, LPS, TNF-α and TGF-β1) at several levels of the fibrosis AOP [29] provided convincing evidence supporting that the described sequence of cellular events could be reproduced *in vitro*.

The application of such a system would be a great contribution for the further understanding of the mechanism through which clinically relevant compounds lead to liver fibrosis. The implementation of this system for the detection of potential pro-fibrotic compounds may replace currently used animal studies. In addition, the model system allows testing of anti-fibrotic compounds in a physiological relevant *in vitro* system, providing researchers a novel tool to study inhibition of fibrosis progression.

## Abbreviations

AOP: Adverse outcome pathway
ATP: Adenosine triphosphate
BSA: Bovine serum albumin
CD44: Cluster of Differentiation 44
CD68: Cluster of Differentiation 68
Col1α1: Collagen 1 alpha 1
Col1α2: Collagen 1 alpha 2
Col4α1: Collagen 4 alpha 1
DMSO: Dimethyl sulfoxide
ECM: Extracellular matrix
FBS: Fetal bovine serum
GOI: Gene of interest
H&E: Haematoxylin and eosin stain
HBV: Hepatitis B virus
HCV: Hepatitis C virus
HSC: Hepatic stellate cells
IL1β: Interleukin 1 beta
IL6: Interleukin 6
KC: Kupffer cells
Keap1: Kelch-like ECH-associated protein 1
LPS: Lipopolysaccharide
MMP2: Metalloproteinase 2
mRNA: messenger RNA
MT: Microtissue
MTX: Methotrexate
Nrf2: Nuclear factor E2-related factor 2
PBS: Phosphate buffer saline
PCR: Polymerase chain reaction
PDGF: Platelet-derived growth factor
PFA: Paraformaldehyde
ROS: Reactive oxygen species
SD: Standard deviation
S.E.M.: Standard error of the mean
TAA: Thioacetamide
TGF-β1: Transforming growth factor beta 1
TNF-α: Tumour necrosis factor alpha
αSMA: alpha-smooth muscle actin
